# Patient Opinion of Visiting Therapy Dogs in a Hospital Emergency Department

**DOI:** 10.3390/ijerph17082968

**Published:** 2020-04-24

**Authors:** Joanne Reddekopp, Colleen Anne Dell, Betty Rohr, Barbara Fornssler, Maryellen Gibson, Ben Carey, James Stempien

**Affiliations:** 1 Faculty of Education, University of Regina, Regina, SK S4S 0A2, Canada; jvr603@usask.ca; 2 Department of Sociology & School of Public Health, University of Saskatchewan, Saskatoon, SK S7N 5A5, Canada; colleen.dell@usask.ca; 3 College of Medicine, University of Saskatchewan, Saskatoon, SK S7N 5A5, Canada; betty.rohr@usask.ca; 4 School of Public Health, University of Saskatchewan, Saskatoon, SK S7N 5A5, Canada; barb.fornssler@usask.ca; 5 Department of Sociology, University of Saskatchewan, Saskatoon, SK S7N 5A5, Canada; ben.carey@usask.ca; 6 Emergency Department, Royal University Hospital, Saskatoon, SK S7N 5A5, Canada; stempien@islandnet.com

**Keywords:** therapy dog, emergency department, patient opinion, companion dog, public health

## Abstract

To date there have been no studies examining whether patients want emergency department (ED) therapy dog programs. This patient-oriented study examined the opinions of patients about whether they would want to be visited by a therapy dog in the Royal University Hospital ED. Cross-sectional survey data were collected over a six week period from a convenience sample of 100 adult patients who had not been visited by a therapy dog in the ED. Most (80%) indicated they would want a visit by a therapy dog as an ED patient. A higher proportion of individuals who currently have a pet dog (95%) or identify as having lots of experience with dogs (71%) were more likely to indicate this want compared to those without a dog (90%) or little to no experience with dogs (62%). The majority were also of the opinion that patients may want to visit a therapy dog in the ED to reduce anxiety (92%) and frustration (87%) as well as to increase comfort (90%) and satisfaction (90%) and to a lesser extent to reduce pain (59%). There was no significant difference in findings by gender or age, other than a higher proportion of older adults and females identifying cultural background and tradition as a possible reason that patients may not want to be visited by a therapy dog. The findings of this study can help guide considerations for future ED therapy dog programs.

## 1. Introduction

The number of companion animals in Canadian households has increased over the past decade with approximately 41% of households containing a dog and 37% a cat [1]. While the cat population has stabilized over the past several years in Canada, the dog population has increased [1]. Emerging research has indicated that interacting with companion animals can have a positive effect on humans, including improved psychological, emotional, and physical health [2,3,4,5]. A recent review of the literature by Hodgson et al. identified four key benefits of pet ownership to human health: “as builders of social capital, as agents of harm reduction, as motivators for healthy behavior change, and as potential participants in treatment plans” [2] (p. 526). They refer to the benefits to human health from interacting with animals, focusing on companion animals, as zooeyia [6].

Therapy dogs have been gaining acceptance as a complementary health intervention over the past decade in a variety of health care settings. Therapy dogs are companion animals who volunteer alongside their human handlers to offer other people a brief (approximately 10 min) visit in a setting where individuals do not typically have access to a pet [7]. This is referred to as “being visited” by a therapy dog in this paper and is commonly referenced in the literature as an animal assisted activity [7]. There are no professional therapeutic goals identified for these visits, but the interaction may be considered therapeutic [5,8]. Handlers and their therapy dogs who volunteer in infirmary-related animal assisted activities commonly visit nursing facilities, palliative care wards, psychiatric units, surgical wards, and rehabilitation centres [8,9,10]. Research evidence is indicating that being visited by a therapy dog can have a positive effect on reducing patient stress, pain, anxiety, and anger [5,9,11,12,13,14,15]. Research is likewise demonstrating that therapy dog visits can provide participants with a sense of comfort and support [16,17,18,19,20]. Emergency department (ED) patients commonly experience distress in a hospital ED, experienced as “a mix of anxiety and depressive symptoms” [21]. A recent US study found that being visited by a therapy dog was accepted by staff and patients in an ED environment [22].

Despite the documented benefits of animal assisted activities in hospital and other settings, researchers have criticized the emerging field for its lack of methodological rigor. Studies have also disproportionately focused on aging populations and have paid limited attention to the impact of gender. Researchers have suggested the need for increased research on therapy dog visiting programs generally, including in the novel area of hospital ED visits [14,15,23,24,25,26,27,28].

Building from Dell et al.’s Canadian study of the experiences of patients visited by a therapy dog in the Royal University Hospital (RUH) ED, the current study aims to provide empirical evidence of patient opinion on desire to be visited by a therapy dog in the ED and why or why not [14]. Further, this study aims to better understand the characteristics of patients who are supportive and nonsupportive of therapy dog visits, including age, gender, and dog ownership experience. A patient-oriented research approach that prioritizes patient, as well as health care worker, views throughout the research process was applied in this public health study. There is a growing call to recognize the patient experience as an important contributor to quality healthcare in both practice and research [29,30,31,32].

## 2. Methods

### 2.1. Setting

The RUH is a major teaching hospital connected to the College of Medicine at the University of Saskatchewan and was the first ED in Canada to offer an animal assisted activity (i.e., therapy dog program). It is the trauma and tertiary care centre for the province. The ED is a 24-hour service and is the busiest ED in the province. It is one of three adult and one children’s hospital in the city of Saskatoon. It is part of the Saskatchewan Health Region, a complex and integrated health delivery agency for the province.

The first therapy dog from the St. John Ambulance Therapy Dog program visited the RUH ED in January 2016. The goal of the St. John Ambulance therapy dog program coincides with that of the organization—to offer charitable, humanitarian care to the sick and injured [33]. The therapy dog program aims to offer support and comfort to individuals with whom the dogs visit [34]. This goal coincides with the mandate of the Saskatchewan Health Authority, which is to improve health and well-being with a commitment to patient- and family-centred care [35].

### 2.2. Participants

A convenience sample of 100 adult patients (18 years of age and older) in the RUH ED waiting room were recruited to take part in the study by the lead author of this paper and a research assistant. This sample size was chosen with the aim to obtain a snapshot of patient views as well as for appropriate statistical comparison of two groups using two proportions z-test and two independent sample t-tests [36]. All participants had registered with triage and were approached when waiting in the general ED area to be seen by public health care staff. On average, it took six minutes for participants to complete the questionnaire. To complete it, participants could enlist the assistance of friends or family who accompanied them to the hospital but were instructed that the answers provided were to be from their own perspective. No identifying information was collected to maintain participant confidentiality.

Fifty-eight of the 100 participants identified their gender as woman and the remainder (42) as man, and no gender nonbinary identities were reported in the fillable gender response area. The average participant age was 44 years, with a range from 21 to 80 years. For analysis, the sample was divided into three equally sized groups, 32% of participants were 35 years of age or younger, 33% of participants were 36 to 49 years of age, and 32% of participants were 50 years of age or older. The data shows that 38% of participants currently have a dog as a pet, slightly over the national average [1]. Sixty-six percent of all participants also indicated that they have a lot of experience with dogs.

### 2.3. Study Design

This study was designed as a patient-oriented cross-sectional survey. All participants were recruited in the English language and none were non-English speaking. Individuals in critical condition or who were actively engaging with a public health care professional were not approached so as to not interfere with their medical care or add an undue burden. Data collection occurred prior to a therapy dog visiting the hospital on the data collection day. We chose this because we wanted to separate the potential impact of the therapy dog from the data being collected. Research shows, for example, that visiting with a dog for just five minutes can increase human oxytocin (i.e., feel-good hormone) levels [5,9] and this could positively skew the questionnaire responses. Data were collected on various days of the week and times of the day (morning, afternoon, and evening) over an approximate one month period, between 15 February 2018 and 27 March 2018. The lead researcher attended the ED 3 or 4 times a week and for an approximate 3 hour period each time. A research assistant trained by the lead researcher collected data on three occasions. Human Ethics Board approval was granted from the University of Saskatchewan and the Saskatchewan Health Authority. All participants signed a consent form to participate in the study.

### 2.4. Measure

A questionnaire was used to collect the data and was comprised of four sections. Basic demographic information for the participants (gender and age) was gathered to determine the sample population composition. Second, data on pet ownership was collected (experience with dogs; currently have a dog; experience with pets other than dogs). Open-ended questions were included for possible participant elaboration (i.e., do you have any comments to add about this experience). The third section asked the participants a question about their knowledge of therapy dogs (i.e., have you heard about therapy dogs visiting in hospitals), and a definition was provided (a therapy dog is a dog who visits schools, hospitals, and nursing homes with its owner as a volunteer). The opinion section of the questionnaire was divided into two subsections: reasons people may want and reasons people may not want a visit with a therapy dog as an ED patient. Likert-type responses (1 = strongly disagree to 5 = strongly agree) were drawn from an in-depth review of the relevant empirical literature that highlighted reasons individuals may or may not want a visit by a therapy dog [5,6,9,37]. We chose the nine most common reasons because of the desired brevity of the questionnaire in the ED setting. We also included room for additional responses not listed (i.e., are there any other reasons you can think of that are not listed above). A final question was asked in this section about the participant’s desire for a visit by a therapy dog in the ED if given the opportunity (i.e., If you had the opportunity to visit with a therapy dog in the emergency department today, would you want to? Why or why not?).

The questionnaire’s use of a five-point Likert-type scale to collect the data is a common and accepted means for measuring participant opinion [37]. To increase the reliability of the two targeted domains in the questionnaire (i.e., reasons people may want a visit by a therapy dog or not), participants were given 4 or 5 reasons to choose from for why they think patients may or may not want a visit by a therapy dog. To control for an acquiescence response bias, the Likert-type questions were written in both positive and negative formats [37].

The questionnaire was designed to be understandable at a fifth-grade reading level to ensure a broad audience would be able to answer the questions presented. It was reviewed by patient advisors on the study team to ensure face validity before implementation in the ED. The lead researcher on the study is an emergency paramedic with over 15 years of experience with patients in the RUH ED, and her intimate knowledge of the ED and patients helped to guide the patient-oriented research design of the study.

### 2.5. Data Analysis

Data analysis were conducted to describe the sample and to examine differences between gender (men and women) and age groups (≤35 years old, 36–49 years old, ≥50 years old). Two population proportion z-tests were used to determine if there were differences in the group proportion on a categorical characteristic. Independent sample t-tests were conducted to determine the difference in group means. Additionally, mean differences within gender and age were calculated to see if perceptions of therapy dogs in the ED differed. Participant responses of their desire to have a visit by a therapy dog were examined by history of dog ownership and dog experience to determine if previous dog interactions influenced desire for a therapy dog visit [6].

## 3. Results

### 3.1. Opinion of Therapy Dogs Visiting in the ED

A large majority (80%) of the study participants indicated they would want a therapy dog visit in the ED if it was available. Many study participants agreed or strongly agreed that patients may want to visit a therapy dog in the ED to reduce anxiety (92%), reduce frustration (87%), increase comfort (90%), and increase satisfaction (90%). Mean values for the reasons one may want to visit a therapy dog ranged from 4.1 to 4.3 (where a 4 rating was “agree” and a 5 rating was “strongly agree”). Over half of participants (59%) agreed or strongly agreed that other patients may want a visit by an ED therapy dog to reduce their pain (M = 3.7). Additional responses participants offered for possibly wanting a visit by a therapy dog in the ED included: as a distraction (e.g., cute, relieve boredom) (6%); to offer companionship and affection (4%), and to bring happiness (3%).

Participants were asked in the questionnaire the degree to which they agreed a patient may not want to have a visit by a therapy dog in the ED because of fear or allergies, and a strong majority agreed—85% allergies, 82% fear. Mean values were 4.0 and 3.9, respectively. Forty percent of the participants agreed or strongly agreed that patients may not want to visit a therapy dog because of cultural background or tradition (M = 3.2). Relatively fewer participants (15%) agreed or strongly agreed that patients may not want to visit due to the potential for zoonotic (animal to human) disease transmission (M = 2.2). Additional responses given for patients potentially not wanting a therapy dog visit included: the sanitary environment of a hospital and dogs being unsanitary (e.g., shedding) (4%); potential harm to the dog (2%); and dislike or no experience with dogs/like cats (3%).

#### 3.1.1. Opinion Variation by Gender

On average, women were statistically significantly higher in agreement than their male counterparts that patients may not want a visit by a therapy dog in the ED due to cultural background or tradition (Mean difference 0.53, *t*(1.98) = 2.87, p = 0.005, and Effect size Cohen’s d = 0.57) (Table 1). 

#### 3.1.2. Opinion Variation by Age

A higher proportion of the “50 and over” age group (63%) was in agreement that patients may not want to visit a therapy dog in the ED due to cultural background or tradition compared to their counterparts (28% of “35 or lower” and 30% of “36 to 49” age groups) (Table 2). This finding was statistically significant (Proportion difference of 35%, z = 2.81, p = 0.005, Effect size Cohen’s h = 0.72 and Proportion difference of 33%, z = 2.67, p = 0.008, Effect size Cohen’s h = 0.67).

### 3.2. Opinion Variation by Dog Ownership

The data shows that 38% of participants currently have a dog as a pet and 66% also stated that they have a lot of experience with dogs. A significantly higher proportion of the study participants that currently have a dog as a pet (95%), compared to those that do not (71%), indicated they would have a therapy dog visit them in the ED if it was offered (z = 2.92, p = 0.0035, Effect size Cohen’s h = 0.69). In addition, a significantly higher proportion (90%) of those who self identified as having lots of experience with dogs indicated they would want to have a therapy dog visit in the ED compared to those with some or no dog experience (62%) (z = 2.92, p = 0.0035, Effect size Cohen’s h = 0.69).

## 4. Discussion

The majority of participants were of the opinion that patients may want a visit by a therapy dog in the ED to reduce anxiety and frustration and to lessen pain, as well as increase comfort and satisfaction with their ED experience. A 2019 study in the RUH ED concluded that participation in a therapy dog intervention improved patients’ comfort, decreased unpleasant and distressing emotions, and provided a helpful distraction from the ED setting [14]. This is synchronous with the general ED patient experience literature which indicates that patients commonly experience the ED as a stressful environment. This is due to such ED experiences as overcrowding, delayed pain management, long wait times, and troublesome emotions (e.g., anxiety) with an unknown diagnosis [31,32,38,39]. Researchers have found that a supportive and caring hospital environment can reduce patient stress and anxiety [40,41,42]. Therapy dog research has indicated that being visited by a therapy dog in a stressful environment, such as a hospital, can support positive emotions (e.g., joy, love, calmness) among participants [43,44,45,46]. Our current study’s finding is an important addition to the ED literature which seldom forefronts patient opinion in the offering of care [30].

Participant opinions in this study supported concerns found elsewhere in the literature for why a patient may not want a visit by a therapy dog in the ED, including allergies, fear, cultural background or traditional reasons, and potential for disease transmission [5,6,9]. Most reasons for why patients may not want a visit by a therapy dog were not remarkably different by gender or age group. The notable differences with medium size effects were that women and older individuals tended to identify cultural background or tradition as a possible reason patients may not want a visit by a therapy dog compared to their counterparts. This supports consideration of the influence of gender and age in future studies and ED therapy dog program considerations. The only other two studies known to the authors that examined the acceptance of therapy dogs in an ED environment concluded that they were accepted but did not examine this by reason or account for the influence of gender and age as the current study did [28,47].

Reasons for the difference in opinion by gender and age on cultural background and tradition as a reason not to be visited by a therapy dog are unknown and require further study. This may include, for example, exploring potential differences in cultural tolerance and awareness by gender and age [48,49]. While a study does not exist, we do know from the case study of the patient wait experience in the RUH ED with therapy dogs that females reported a greater benefit than males after a therapy dog visit [14]. Further, we know that the majority of therapy dog studies have focused on the experiences of visits with seniors and school-aged children [50,51] but have not considered the influence of gender and age.

Related to this, the importance of accounting for age and gender is identified in the pet ownership literature. This is highlighted, for example, in the work of Saunders et al. (2017); they suggest that “pet owners and nonpet owners differ across many traits, including gender, age, race/ethnicity, living arrangements, and income… (and these factors) are related to a range of mental and physical health outcomes” [52] (p. 1). The influence of gender and age should be considered in the unfolding therapy dog research field, including as considerations in the implementation of therapy dog programs in an ED setting. Exploration of the pet ownership literature may provide guidance.

Data collected about the participants’ ownership of a pet indicated that a greater number of individuals currently did not own a pet dog compared to those that did, and a greater number of individuals indicated they had a lot of dog experience compared to those that indicated they did not. Reflecting on their own opinion about being visited by a therapy dog in the ED as a patient, the majority of participants indicated they would want a visit. Individuals who currently had a pet dog or have experience with dogs were more likely to want to be visited by a therapy dog in the ED than those who did not. While research into this area is lacking, reasons for this difference may include that those with a pet dog and/or with extensive dog experience simply like dogs more, are more acutely aware of the comfort dogs can offer because they live or have lived with one, and for some, have personally experienced pain and anxiety reduction related to an illness because of their pet. As another consideration, Crossman et al. found that people who hold positive perceptions of companion animals are more likely to positively perceive animal related interventions than those with negative attitudes toward pets [53]. A clinical trial in the United States demonstrated that canine visits reduced patient perception of anxiety in an emergency care setting but did not account for pet ownership and experience [15]. Once again, further research is required.

Having the opinions in this study originate from the patients’ perspective supports a patient-centred approach to public health in an ED setting. There is a need for further patient-oriented research in the therapy dog and ED field. There has historically been a dearth of attention to accounting for the patient experience in health care, though this is changing [29]. The Canadian Institutes of Health Research is a major health research funding body in Canada and in the past decade it has developed a national strategy for patient-oriented research [54]. Therapy dogs are a discernable area to apply a patient-oriented approach, and as this study did, starting with the foundational question of whether patients want to be visited by a therapy dog, and why or why not. 

## 5. Limitations

There are several main limitations to this study. First, the survey was only completed in the English language and excluded the views of patients under the age of 18 years. Although all participants were English-speaking, some may not identify English as their primary language and not be as fluent. Eighteen years was considered the minimum age of inclusion because the RUH ED does not provide care to those under 18 years of age. We also did not ask a question on race/ethnicity. Next, patients self-selected out of participating in the study and it was not determined if they declined due to pre-existing concerns with dogs/therapy dogs, so it is possible perceptions of reasons not to have a visit by a therapy dog were missed. In addition, patients admitted to the ED as high priority, critical care patients were not accessed so as not to impede on their medical care. Fourth, potential for sampling bias and sampling error is inherent to a convenience sample. Fifth, patients were asked reasons people would want to have a visit by a therapy dog or not in the ED. The question was worded in this way to capture the public nature of the ED setting, asking about their individual opinion as well as others’. A limitation of this approach is that it can capture stereotypical opinions of other patients. Sixth, it is unknown if the demographics of the sample in this study mirror the typical ED client population. For example, it is unknown if the low recognition of cultural background or tradition as a reason for not wanting to have a visit by a therapy dog reflects an opinion representative of the RUH ED population. Further, we did not have any participants identify with a nonbinary gender identity. Next, to have the questionnaire brief enough for an ED environment, only the top five reasons for wanting to have a visit by a therapy dog in the ED and the top four for not were chosen from the literature. Finally, despite attempts to control for participants’ interaction with a therapy dog, it is possible patients may have had contact with a therapy dog at a prior ED visit and it was unknown to the data collection team. This may influence the participants’ responses.

## 6. Conclusions

The opinion of the majority of study participants was that they would like to have a visit by a therapy dog in the ED and perceived other patients to want to have a visit as well. Individuals who currently have a pet dog or have experience with dogs were more likely to indicate this compared to those without a dog or dog experience. The majority were also of the opinion that patients may want to visit a therapy dog in the ED to reduce anxiety and frustration, as well as to reduce pain and increase comfort and satisfaction. There was no significant difference in findings by gender or age, other than older adults and females identifying cultural background and tradition as a possible reason to not want to have a visit by a therapy dog. With no patient-oriented study ever undertaken to determine whether therapy dog programs are desired by patients in an ED setting, these findings can help guide considerations for future ED therapy dog programs.

## Figures and Tables

**Table 1:** Proportion of participants that agree/strongly agree and mean for reasons patients may not want to have a visit by a therapy dog in the emergency department (ED): Gender.

Gender	W				M				Total			
Reason	N	% Agree	M	SD	N	% Agree	M	SD	N	% Agree	M	SD
Diseases	57	16	2.2	1.13	41	15	2.1	1.14	98	15	2.2	1.13
Allergic	58	88	4.1	0.54	42	79	3.9	0.84	100	85	4.0	0.69
Cultural	58	47	3.4 *	0.84	42	31	2.9	1.02	100	40	3.2	0.95
Afraid	54	85	4.0	0.61	35	77	3.8	0.73	89	82	3.9	0.67

* Significantly higher p < 0.05.

**Table 2:** Proportion of participants that agree/strongly agree and mean for reasons patients may not want to have a visit by a therapy dog in the ED: Age.

Age Group	35 or Lower				36 to 49				50 or Higher			
Reason	N	% Agree	M	SD	N	% Agree	M	SD	N	% Agree	M	SD
Diseases	32	19	2.4	1.10	32	13	1.9	1.13	31	13	2.2	1.13
Allergic	32	84	4.0	0.54	33	91	4.1	0.61	32	75	3.9	0.91
Cultural	32	28	3.0	0.82	33	30	3.0	1.06	32	63	3.6 *	0.88
Afraid	27	78	3.9	0.70	29	83	3.9	0.62	30	90	4.0	0.69

* Significantly higher p < 0.05.
